# Culturally adapted flowcharts in obstetric emergencies: a participatory action research study

**DOI:** 10.1186/s12884-022-05105-z

**Published:** 2022-10-14

**Authors:** Estefanía Bautista-Valarezo, María Elena Espinosa, Nele R. M. Michels, Kristin Hendrickx, Veronique Verhoeven

**Affiliations:** 1 Health Sciences Department, Private Technical University of Loja (Universidad Técnica Particular de Loja, UTPL), San Cayetano alto s/n, 1101608 Loja, Ecuador; 2 Department of Family Medicine and Population Health, School of Medicine and Health Sciences, University of Antwerp, Universiteitsplein 1, 2610 Antwerp, Belgium; 3 Programa de Doctorado en Ciencias Médicas, Universidad de la Frontera, Temucho, 4811230 Chile

**Keywords:** Flowcharts, Obstetric emergencies, Traditional midwives, Pre-eclampsia, Shoulder dystocia, Postpartum hemorrhage

## Abstract

**Introduction:**

Maternal mortality is a health problem in developing countries and is the result of several factors such as sociodemographic and economic inequalities and difficulties in accessing the health services. In addition, training strategies in obstetric emergencies targeting the non-medical personnel such as traditional midwives are scarce. The focus of this study is to develop learning and communication bridges on the management of obstetric emergencies and on policies of patients’ referral to the biomedical health system in rural areas.

**Methodology:**

A Participant Action Research (PAR) study with a mixed methods approach was set up to elaborate culturally adapted flowcharts. The project lasted approximately 3,5 years, from September 2016 to January 2021.

**Results:**

The study was conducted with 94 traditional midwives from southern Ecuador and is divided into 4 phases, namely: 1) *Exploration*: focus groups and interviews were conducted to document the management of obstetric emergencies through the presentation of “clinical case” scenarios in three important topics, namely: pre-eclampsia, shoulder dystocia and postpartum hemorrhage, 2) *Planning*: a number of reflective sessions were conducted between the researchers and the healers/midwives to elaborate flowcharts. 3) *Action*: the training was conducted in rooms dedicated to proficiency in the aforementioned topics and using the flowcharts, 4) *Evaluation*: 90% of the participants reported having used the flowcharts during the first year after the training. The most frequently used flowchart was that of pre-eclampsia for the recognition of warning signs during pregnancy control.

**Conclusion:**

This study documents common practices of pregnancy and delivery management by traditional midwives. Furthermore, cultural flowcharts were developed for and together with midwives to improve the clinical response to obstetric emergencies. The preliminary evaluation was favorable; the most frequently used flowchart concerned preeclampsia. In this process, establishing a partnership was crucial for successful intercultural collaboration.

## Introduction

In 2015, the difference in the maternal mortality rate between developing countries (239 for every 100,000 live births) and developed countries (12 for every 100,000 live births) was enormous [1]; these data reflect the existing inequalities in quality of care and in access to health services. Certain characteristics such as poverty or living in rural areas are the main causes of inequality in developing countries [2]. Another key element is ethnicity, where indigenous peoples are a vulnerable group as they gather all these characteristics in a single context [1].

The “Improving Maternal Health” Goals Report (United Nations) states that a 37% reduction in maternal mortality has been recorded since 2000 and that the percentage of women who have undergone prenatal care has increased. Despite these data, only half of the women receive the recommended share of medical care in developing countries, with mortality still being 14 times greater in these countries than in developed regions [3, 4].

Traditional midwives promote maternal-child health in rural communities and, especially, in places with indigenous populations. They offer care during pregnancy and 40 days after delivery, with the objective of ensuring the mother’s and the child’s well-being. In countries where the maternal mortality rates are still high, projects are designed that focus on saving lives, and increasing care coverage. Within their health plans, some Latin American countries include integration of interculturality, which, in the last 2 decades, has allowed for the reemergence of traditional medicine in the health system. However, ensuring accessibility and availability regarding good quality maternal-child care in rural areas is still a challenge [5, 6].

In order to improve quality of care and to ensure accessibility to the health system, the ideal situation is to work in collaborative teams, including biomedical health staff and traditional health staff. The traditional midwives’ role is providing community care and getting people closer to the biomedical health system. To reach this goal, it is important to offer both parties, biomedical and traditional health staff, the necessary tools to attain their objectives [7, 8]. One of the tools is education based on community needs, where midwives are able to make decisions and improve care coverage. The creation and implementation of culturally-adapted algorithms in obstetric emergencies, for example supporting medical and referral making, comply with all of the aforementioned proposals: as such, they can become a strategy that can assist in reducing maternal mortality and improving the quality of the care provided to the mothers and children [9].

In developing countries, the obstetric emergencies with the highest maternal mortality rates are pre-eclampsia, postpartum hemorrhage and childbirth complications [1]. A number of strategies have been implemented to reduce the risk of maternal death due these three complications. Clear examples are the observational studies conducted by Lutgendorf et al. [10] and Dillon et al. [11], which evaluated clinical performance and the results associated with postpartum hemorrhage after the implementation of a multidisciplinary simulation program, concluding that training was associated with a better clinical response [10, 11].

This study focused on the training of traditional midwives from southern Ecuador in obstetric emergencies by means of culturally adapted flowcharts next to training in the form of simulation sessions. In this way we intend to improve the care provided to pregnant women and women post-partum in indigenous communities and additionally link all the actors involved in the community context.

### Methodological design

Participant-Action Research (PAR) was set up for this purpose. In this study, we sought to create reflection, dialog, action and learning bonds between the traditional midwives and the biomedical personnel. PAR seeks to enable participatory communities to understand and reflect about the problems they experience and promote actions to improve their living conditions [12, 13].

The study was conducted in rural areas from southern Ecuador (Loja/“Saraguro”, Cuenca) where traditional midwives from the Quechua nationalities live, and which are important places for community meetings and traditional ceremonies. This study was developed in four stages: exploration, planning, action and evaluation. In this study, PAR was used to develop learning and communication bridges through flowcharts on the management of obstetric emergencies and patients’ referral to the biomedical health system in rural areas. The project lasted approximately 3,5 years, from September 2016 to January 2021.

### Ethical approval

The ethical requirements set forth by the Research Committee of the San Francisco de Quito University were fulfilled and the Ministry of Public Health approved the study (CEISH USFQ 2017-059E). Written consent was obtained from the indigenous community (the “Saraguro and Cuenca” healers’ council) and from the participants, who were free to decline or withdraw their participation at any time.

### Sampling

Convenience sampling was used to recruit the participants. During the initial research phase, a workshop was held where the project was presented, and it was indicated that in the final phase, each participant would receive instruments? according to the project’s objective and the needs of each healer. The traditional midwives who agreed to participate in the research did so voluntarily. The inclusion criteria were as follows: working as a traditional midwife and speaking Spanish. However, the participants’ mother tongue is not Spanish, which explains the grammatical errors found in the quotes.

### Phases of participant action research

#### Phase 1 - exploration

In this exploration phase, we used two data collection instruments. Focus groups were used at a first moment, which allowed for an initial reflection and interaction with the participants about the theme proposed [14]. Subsequently, semi-structured interviews were conducted, which allowed us to go deeper into data that had not been discussed in the focus groups, and to introduce emerging categories [15, 16]. The guide used to develop the focus groups and the interviews consisted of three clinical cases which clearly presented the theme of “obstetric emergencies” (Table 1). The topics were selected according to the main causes of maternal deaths or complications during pregnancy, delivery and postpartum at the local and national levels; the topics which appear most frequently in statistics are pre-eclampsia, postpartum hemorrhage and shoulder dystocia; these were chosen for the present study. Next to specific information on dealing with emergencies, some general themes on pregnancy counseling were brought up by our participants during the focus groups. A total of 9 focus groups were conducted with 94 participants aged between 20 and 70 years old (Table 2). The focus groups lasted between 65 and 140 minutes plus 30 minutes for initial introduction. After that, 12 interviews lasting from 60 to 90 minutes were conducted (Table 3). Informed consent and authorization to audio-record the discussions were requested. The participants were guaranteed confidentiality of their contributions.

**Table 1:** “Obstetric Emergencies” clinical cases

**CLINICAL CASE 1: pre-eclampsia**	
A 35-year-old patient who gave birth through a normal delivery 4 years ago; she is currently pregnant with 38 weeks (9 months) of gestational age; she asks for your service because she had delivery-related pain yesterday, which has become very frequent now. She then evolved to normal delivery. She had a male child that looks big and heavy.	
Half an hour ago, while lying in her bed, it is noticed that she presents abundant bleeding, that she wets the bed, feels weak, looks sweaty and pale, and says that her heart is beating faster.	
- What would you do in this situation?	
- What would you do with Juanita?	
- What would you look at (watch) in the patient?	
- What do you think causes the bleeding?	
- What would you do with Juanita to stop the bleeding?	
- Would you subject her to any exam?	
- What would you do with Juanita if the bleeding does not stop?	
- Do you ask for help? To whom, and why?	
**CLINICAL CASE 2: postpartum hemorrhage**	
Primigravida patient with 9 months of gestational age, she has been having contractions for some days; but they have gained strength and become more painful now. She also says that her water broke approximately 2 hours ago. You ask her to lie down to examine her, and notice that the child is coming out. You ask her to push, the child’s head comes out, but something goes wrong and the rest of the body is stalled inside.	
- How long would you wait for the child to come out?	
- How do you realize that the child is not going to come out?	
- What would you do in this situation?	
- If the child doesn’t come out whatever your efforts, what would you do?	
- Do you ask for help?	
**CLINICAL CASE 3: shoulder dystocia**	
Pregnant patient (9 months), you have been monitoring her. She comes to you today because she says that she feels unwell, tired, with a headache, and that she has seen little white lights in the morning. She also says that her hands and feet are too swollen these last days. She doesn’t have delivery-related pain.	
What do you think of this case?	
Would ask anything to the patient?	
What would you do in this situation?	
Would you subject her to any exam or test?	
What do you think is the patient’s problem?	
What would you do for Norma to get better?	
Do you ask for help?

**Table 2:** Focus groups

Code	Place	Ethnicity	Number of participants
FG1	Loja	Kic, Sh, M	10 traditional healers
FG2	Loja	Kic, Sh, M	11 traditional healers
FG3	Cuenca	Kic, M,	11 traditional healers
FG4	Loja	Kic, Sh, M	11 traditional healers
FG5	Cuenca	Kic, M	10 traditional healers
FG6	Cuenca	Kic, M	10 traditional healers
FG7	Loja	Kic, Sh, M	10 traditional healers
FG8	Loja	Kic, Sh	10 traditional healers
FG9	Cuenca	Kic, M	11 traditional healers

*Kic* kichwa, *M *mixed race, *Sh* shuar

**Table 3:** Interviews

Code	Place	Ethnicity
I1	Loja	Kic,
I2	Loja	Sh
I3	Loja	M
I4	Cuenca	Kic
I5	Loja	Kic
I6	Loja	Kic,
I7	Loja	Sh
I8	Loja	M
I9	Cuenca	Kic
I10	Loja	Kic
I11	Cuenca	Kic
I12	Cuenca	Kic

*Kic* kichwa, *M *mixed race, *Sh *shuar

A thematic analysis with a phenomenological approach was used in this part of the study. This method allowed us to identify the essence of the phenomenon, as well as to obtain the participants’ options and ideas in a reflective manner [17].

The data recorded from the focus groups, the interviews and the observation notes were transcribed verbatim and analyzed with the N-Vivo software. The researchers reviewed the data and assigned codes. The code book was shared, analyzed and interpreted by the researchers. In addition, categories and subcategories that describe the essence of the problem, and the context were identified.

#### Phase 2: planning (creation of a plan): creation of flowcharts

By means of reflective sessions the results, obtained in the exploration phase, were presented to the traditional midwives and flowcharts about obstetric emergencies were developed. In this way, flowcharts (*N* = 3) on the following topics were created: a) pre-eclampsia, b) postpartum hemorrhage, and c) shoulder dystocia. Reflexivity in data collection, analysis and interpretation allowed addressing some problems and establishing tools (flowcharts) that enable better care for women before, during and after delivery, recognize warning signs and refer the patient to the biomedical health system in case of any obstetric emergency [18].

The midwives’ involvement contributed to improving the tools / flowcharts through effective feedback and empowering the research participants. Finally, these culturally adapted flowcharts were designed and presented again to the traditional midwives; last changes were implemented following several suggestions, after which the culturally adapted flowcharts were accepted by all participants.

#### Phase 3: action plan

Workshops based on the culturally adapted flowcharts developed in phase 2 were planned for the healers/midwives from the Saraguro and Cuenca communities. The objectives were as follows: to develop skills and competencies in the management of obstetric emergencies (pre-eclampsia, postpartum hemorrhage, and shoulder dystocia), to recognize warning signs, and to refer the patient to the biomedical health system in a timely manner in case there was any sign of emergency. In addition, it was sought to build communication bridges between the midwives and the biomedical system to improve the work performed at the community level.

### Workshops

The workshops were initiated with presentation of the same clinical cases used in the focus groups; subsequently, the culturally adapted flowcharts were presented and skills were practiced with models and manikins. Finally, the second part of the practice was performed in culturally-adapted delivery rooms where knowledge was shared between the midwives and the researchers. A total of 94 traditional midwives attended the training workshops. In addition, as required by the Ecuadorian Ministry of Public Health, 60 midwives from other communities in southern Ecuador were trained.

#### Phase 4: evaluation

In the fourth phase, traditional midwives’ use of culturally-adapted flowcharts in obstetric emergencies were assessed on year after training. The assessment instrument was a survey to examine the process of knowledge construction in the participants.

The parameters evaluated were the following: 1) skill learning and performance of the midwives for the management of obstetric emergencies; 2) recognition of warning signs in obstetric emergencies, and 3) referral to the hospital or to the biomedical health system.

### Reflexivity

Research team was multidisciplinary and consisted of MEBV, MEEG, NM, KH and VV. NM, KH and VV are GPs and senior researchers at the department of Family Medicine and Population Health of the University of Antwerp (UA), Belgium. The project was developed in the context of a partnership between UTPL and UA. All members of the research team have a background in the biomedical health system.

## Results

The total sample consisted of 94 participants. 78% of them belong to the indigenous ethnic group (Quechua), 100% speak Spanish, and 48% speak both languages (Spanish and Quechua). Finally, 84% stated having acquired their knowledge as midwives as a legacy from their parents or grandparents (Table 4). The themes that emerged in the focus groups were broader than the policies on emergencies and were classified into 5 categories: 1) pregnancy control (1), obstetric emergencies: incorrect descent of the baby (2), pre-eclampsia (3), postpartum hemorrhage (4), and postpartum control (5) (Table 5).

**Table 4:** Sociodemographic Characteristics

Characteristics	N (%)
**Age**	
< 30	4 (4)
30 - 49	55 (59)
50 - 69	32 (34)
> 70	3 (3)
**Language**	
Quechua and Spanish	45 (48)
Spanish	49 (52)
**Ethnicity**	
Quechua	88 (94)
Mixed race	6 (6)
**Schooling level**	
Illiterate	5 (6)
Elementary School	66 (70)
High School	23 (24)
**Knowledge acquisition**	
Legacy from parents	79 (84)
Academic studies	0 (0)
With the midwife from the community	15 (16)

**Table 5:** Categories about the experience in the resolution of obstetric emergencies

Category	Subcategories
Pregnancy control (1)	
Obstetric emergencies	Incorrect descent of the baby (2) Pre-eclampsia (3) Postpartum hemorrhage (4)
Postpartum control (5)	

### Pregnancy control

The traditional midwives from southern Ecuador monitor the pregnant women throughout their pregnancies. They provide care at the patient’s home or in their own houses. During the control phase, questions are asked about warning signs or symptoms that help them identify complications; their main focus points are vaginal bleeding, absence of movements in the baby, and lower limb edemas. During the physical examination, gestational age and the baby’s position and size are checked through techniques that involve touching the pregnant woman’s abdomen. If the baby is not in the cephalic position, they perform the technique called “*manteada*” to correct its position. This technique involves “placing a bed sheet under the mother and making rocking movements, while the midwives position the baby, if it does not do so itself”; the midwives can also position it by applying massages to the abdominal region (Q1). Surveillance during the last month is important to know if the midwives can assist the delivery or if they must refer the pregnant women to the hospital or other (biomedical) health center (Q2). They also monitor the family as a source of support for the mother.

***Q1-I2:*** *When the child is crossed, then the “guagua” (baby) is all straightened up when it's sitting for it to get into position. I do the “manteada”, and it positions itself alone or I help.*

***Q2-I1:*** *Yes, on the belly I always have the measure with the hands; I know where the head is, I already see where the head is, then I have the measure; they taught me that this is the measure of the little head inside the belly; then, it's just that the patient can give birth and if the little head is bigger she can't give birth either, they need to be taken to the doctor.*

During pregnancy, the midwives recommend a good diet to the mothers, rich in products that they grow themselves. In addition, some of them recommend that the mothers see a physician for an extra control and exams such as pregnancy ultrasound (Q3).

***Q3-I2:*** *When, for example, they come like this the first months I tell them, I recommend that they see a doctor, that they undergo the echo, whatever, how a person feels, then to go to the doctor.*

### Obstetric emergencies: incorrect descent of the baby

During delivery, the traditional midwives state that one of the complications presented by their patients has been incorrect descent of the “*guagua*” (baby), and they attribute it to problems such as nuchal cord. They manage this complication by sliding the umbilical cord over the baby’s head; subsequently, they stimulate it by cleaning it and they cut the umbilical cord (Q4).

***Q4-I3:*** *I mean, the moment they're born you have to remove the cord, remove it because the little baby is all black, all black…then I remove it, untwist it, remove through the head like that and clean the little baby; because you have to clean all that from the little face, through here and there you already cut the cord and we hand it in to the relatives for them to put some clothes on it.*

Another complication mentioned by the midwives was that of “*guagua chupado*” (“retracted baby”), a baby that cannot descend even if its head can already be seen in the vaginal canal. In these cases, the midwives state that the vertical position (vertical delivery) in a squatting position with the mother’s legs wide open favors descent of the baby. If the baby does not descend despite this position, they move the mother to a crawling position. Once the baby descends, they welcome and care for the newborn (Q5).

***Q5-I3:*** *When the “guagua” is retracted, you have to tell the mum to keep pushing and, as the delivery is in a squatting position, the little baby slides off… some other times it won't come out and I tell the mother to get into a crawling position, like children…. And the baby comes out smoothly.*

When the mother presents complications regarding the baby’s presentation, such as breech, some of the traditional midwives prefer to accompany the patient to the hospital and ask for help (Q6).

***Q6-I2:*** *It happened to me here in the hospital, the child was seated, then the doctors helped me and assisted her so the baby could come out*.

### Pre-eclampsia

During pregnancy control, the midwives look for signs or symptoms that allow them to identify warning situations such as pre-eclampsia. Signs and symptoms identified are lower limb edema, intense headache, dizziness and abdominal pain (Q7). Traditional midwives do not measure blood pressure because they are not aware of the technique or because they lack the necessary materials; given the above, it is important that they continuously monitor and investigate signs and symptoms that guide them to a diagnosis of pre-eclampsia. When facing a suspected case of pre-eclampsia, they treat the patient with medicinal herbs and perform daily controls until the symptoms disappear (Q8). In addition, some midwives refer the patients to the (biomedical) health care center. Sometimes they even accompany the patients to the health care center, although this is often marred/overshadowed by the lack of relationship between them and the biomedical health staff. In situations where the symptoms do not resolve, all of them state the importance of immediately referring the patient to the health care center (Q9).

***Q7-I3:*** *I ask the mother if she has a headache, if she's dizzy, then I do say that pressure is high.*

***Q8-I2:*** *We can't, I mean with the headache and dizziness… and she turns red, really red, then you to have to try to lower the pressure as soon as possible; if she doesn't give birth yet and is still with pressure, you have to put some cabbage with ice immediately on the forehead or aloe vera, that's refreshing, and here also on the crown with the lemon, that lowers it somehow and passion fruit waters.*

***Q9-I3:****… If I haven't been able to lower from there I transfer her immediately, I say come on the pressure is high, not low, Jesus, this needs a doctor.*

Some of the medicinal herbs used for the treatment of pre-eclampsia are passion fruit, parsley, pimpernel, white carnations, *granadilla* (both the fruit and the leaves), etc.

### Postpartum hemorrhage

One of the complications that traditional midwives frequently must assist after delivery is postpartum hemorrhage. Some of the signs and symptoms they categorize as warnings are paleness, palpitations, sweating, low energy and somnolence; in addition, they explore the abdomen and check uterine height. They also check the garments for blood stains to approximately know how much blood the patient has lost. The management performed by the midwives to prevent postpartum hemorrhage is immediately after baby and placenta come out. They apply a massage around the entire abdomen with vegetable or animal oil until reducing uterine height below the navel and remain with the patient watching until bleeding stops (Q10). If bleeding does not stop, the midwives continue with the massage and offer herb infusions. Some midwives tie a band around the mother’s abdomen, over the navel, thus preventing the abdomen from getting filled up with blood (Q11).

***Q10-FG2:*** *For the bleeding you have to massage… upwards from the pelvis until around the navel in this direction and see where it is and watch over here or over there. The matrix has to be here in the navel, in the full center way below the navel.*

***Q11-I5:*** *We grab a band or a piece of cloth… only at this level here we tie it and apply very fast massages at the belly level putting little fats we give her plenty of massages.*

Very few of the traditional midwives had an experience where postpartum hemorrhage has not stopped despite the treatment they applied; in those cases, they refer the patient to the (biomedical) health care center or hospital (Q13). When this is needed, the midwives revealed that they did not feel confident enough or even felt certain inferiority towards the health staff; this is due to the fact that their performance with the patient was questioned on several occasions (Q14).

***Q13-I6:*** *Yes, and if the bleeding doesn't stop with that, then immediately look for a car and take her to the hospital, so that the doctors help.*

***Q14-I6:*** *No… I go to the hospital; I accompany the patient to the door… the nurses look at us… There's no trust.*

The herbs for the infusions, used by the traditional midwives, as part of the treatment for postpartum hemorrhage are the following: parsley, orange, spinach, alfalfa, tree tomato, *mulle*, figs, verbena, oregano, and carrot. The objective of these herbs, especially parsley, is to stop bleeding (Q14-15).

***Q15-FG1:*** *Parsley water is good and also fig water, purple or white carrot water, that's also good for all the blood to come out and the matrix to go strong.*

### Postpartum control

During postpartum control, the patients remain 40 days resting (Q16) and the midwives visit them at their homes almost daily, with the objective of caring for the mother and the newborn. Throughout this period, the midwives are in charge of maintaining good nutrition with special warm food options for fast recovery and which promote breast milk production. Until the fifth day postpartum taking a bath with chamomile water is another important aspect of the postnatal care for the genital area. The objective of this bath is the healing process at the level of the genital area; the rest of the body is not exposed to the water and thus not to temperature changes, as the midwives mention that “if the mother gets cold (temperature change), she runs the risk of breastfeeding interruption due to lack of breast milk” (Q17). However, on days 12, 20, 30 and 40 of the quarantine, the mother receives a full bath with chamomile water not only cleaning the genital area (Q18-19). From the fifth until the fortieth day postpartum, the woman is initially bandaged with a woolen fabric and later with a cotton-like fabric, so that the uterus does not descend and to avoid urinary incontinence problems (Q20).

***Q16-IG5:*** *The mums have to take care of themselves for 40 days, so that they don't get sick later and can work in the fields.*

***Q17-FG2:*** *Down here cleaning is with chamomile water, with this you clean before day five.*

***Q18-FG2:*** *If we apply the bandage after delivery but only with a shawl, only with the shawl and the knot remains here or there towards the front so that it doesn't bother; so that the hip gradually normalizes and do anything or apply force any other thing for the matrix to gradually settle later so that there's no urine descent, nothing.*

***Q19-IG4:*** *I clean her well, then we always did everything on her in the house and until day 12 we haven’t made her walk or anything; put her to eat on the little bed on the side like this; they are seated 5 days, from day 5 to day 12, she's bathed on day 12.*

***Q20-FG1:*** *I've always provided care like this, then at day 12 she can already get up and then fully clean herself but the midwife; no, nobody but the midwife from day 12 to 40, I have cleaned her. As usual 12, 20, 30 and 40 with the medicine they do the bath.*

A fundamental component in the postpartum care is the use of medicinal herbs. For postpartum hemorrhage parsley, verbena, alfalfa, and fig are used. For stimulating breast milk production, they provide fennel with brown sugarloaf, carrot, and chamomile. Pain is managed with chamomile, kapok bush, lemon balm, mauve, maidenhair fern, male *gañel*, or herb of grace, the latter with topical application at the abdomen or hip level. In addition, herbs such as Andean blueberry, chamomile, mauves and *guatos* are used while bathing or cleaning the patient. Finally, they treat problems inherent to their culture, such as “*pasada de frío*” (“stone cold”) or “*mal de los nervios*” (“nerves’ disease”) with *matico*, chamomile, valerian, herb of grace, Andean blueberry, rosemary, white carnations, and others.

The diet is mainly based on the consumption of cereals, vegetables, fruits, and meats like chicken; they totally omit pork, at least during the quarantine. It is important to mention that the mothers should not eat reheated food (Q21-24).

***Q21-FG2:*** *For the bleeding, well there, then immediately you'll see; I take out the alfalfa and lemon balm juice and give her the drink with egg whites with the juice really warm; I boil her chamomile water, I put a little bee honey and give her to drink and massage her a little.*

***Q22-FG4:*** *Lemon balm is very good for pain and nervousness, we have a plant called male “gañel”, they call so a plant that's big, a big flower, then it’ male “gañel”. It's very good too.*

***Q23-I1:*** *To remove pain you give the lemon balm juice with a little bee honey then you give her to drink, and also for example I warm the common rue or mugwort on the little heater and put it in the boiler.*

***Q24-I1:*** *The pregnant woman gets nervous, she gets a colic, she catches a cold, I can put some common rue water with chamomile, a “matico”, or anything, that it catches there; as the baby's already been born, it can choke but to have in a “lejito”.*

### Planning and action

**Fig. 1:**
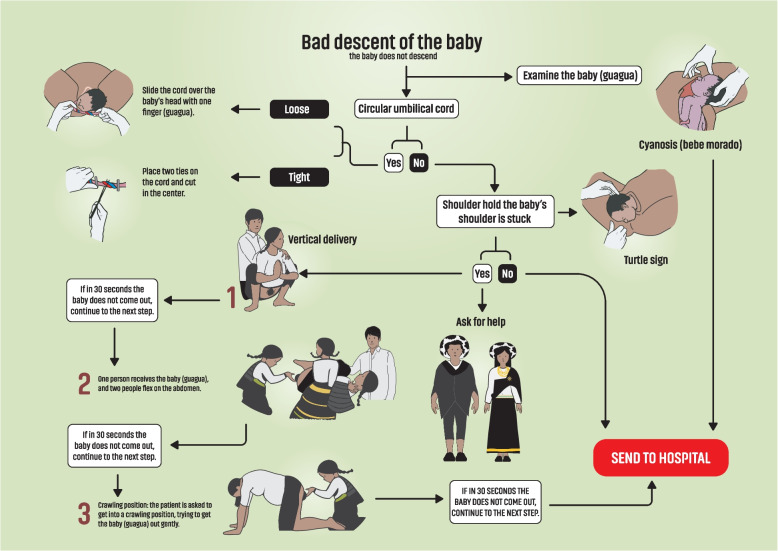
Incorrect descent of the baby

**Fig. 2:**
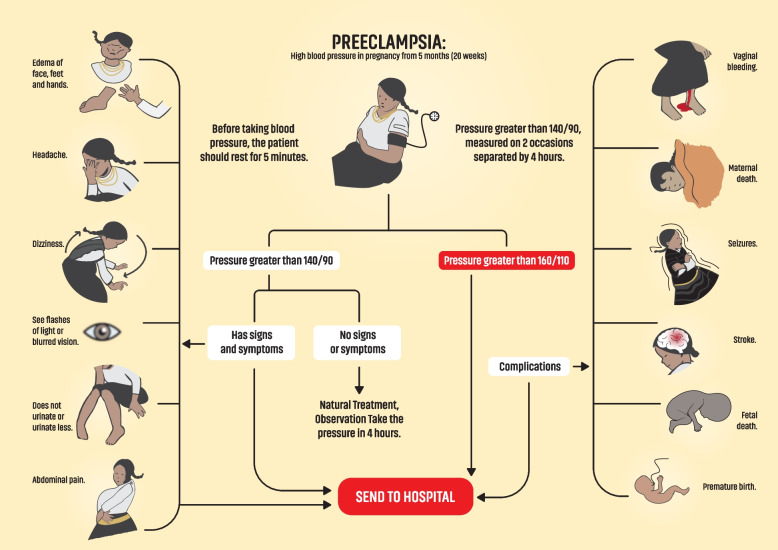
Pre-eclampsia

**Fig. 3:**
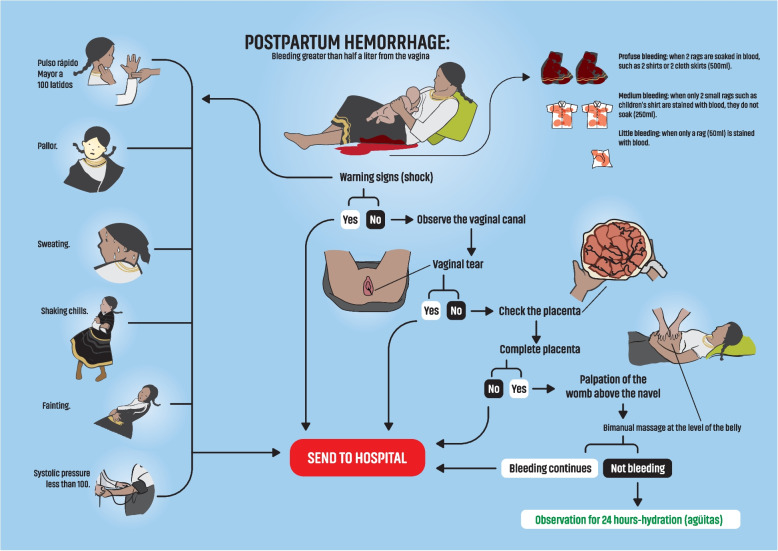
Postpartum hemorrhage

Based on the information obtained from the focus groups and the interviews, the research team met with the traditional midwives in several work sessions and developed culturally adapted flowcharts on obstetric emergencies on the following topics; shoulder dystocia (Fig. 1), preeclampsia (Fig. 2) and postpartum hemorrhage (Fig. 3). The midwives shared not only their expertise in each of the aforementioned fields, but also their experience with the health staff and their ability to work in the biomedical health care system.

The midwives who participated in the project received electronic tensiometers, pulse oximeters, 2-, 4-, and 6-in. elastic and cotton bandages, thermometers, stainless steel scissors and tweezers, sterile glove boxes, and surgical pads. The materials were chosen according to the needs of midwives for the training and care of women during childbirth and obstetric emergencies. Also, the midwives were trained on using the culturally adapted flowcharts and techniques for managing obstetric emergencies (shoulder dystocia, pre-eclampsia and postpartum hemorrhage). For the training, they all received material for the management of obstetric emergencies.

In addition, traditional midwives from other communities were trained thanks to the joint work with the Ministry of Public Health.

### Evaluation

Statistics on emergency obstetric care by midwives are not recorded, because, despite the Ecuadorian Ministry of Public Health (MSP), where registration of care by doctors and healers is mandatory, not all healers working in rural areas report their care to this entity. An estimated number of ERs our participants refer to per year is 2-5, but the current study did not focus on the number of births or patient outcomes. We focused on self-reported knowledge construction rather than patient outcomes in the evaluation.

Firstly, 94 midwives who collaborated in the research were trained; and, secondly, training was offered to 60 traditional midwives from other communities. One year after training an evaluation of the first group was organized. 90% of the participants reported having used the culturally adapted flowcharts. 68% of the participants used the preeclampsia’s flowchart in their practice. They also used the shoulder dystocia flowchart, especially to review the technique required when nuchal cord is present. Ten midwives reported experiencing at least one of the obstetric emergencies. The obstetric emergencies most frequently presented by the patients were postpartum hemorrhage and nuchal cord. Finally, 3 midwives reported that they had to refer their patients to the biomedical health system.

## Discussion

The implementation of culturally adapted flowcharts for the management of obstetric emergencies with the traditional midwives from southern Ecuador was initiated as a strategy to improve maternal/neonatal health and to reduce maternal/neonatal mortality. This has been pursued by strengthening quality of care before, during and after delivery and by addressing obstetric emergencies in a timely manner. Our study was focused on developing culturally adapted flowcharts along with a customized training module to go with each flowchart.

According to data from the Pan American Health Organization, there was an estimated of 303,000 deaths of women during pregnancy, delivery, and postpartum in 2015 in low-income countries [19]. Most deaths occur in low-income countries, with the clear example of Ecuador, which, recorded 180 maternal deaths, 40 more than the number for 2019 (data from the Ministry of Public Health). The main causes were hypertensive disorders (32.52% of the cases), obstetric hemorrhages (19.01% of the cases), and indirect causes (34.35% of the cases) [20]. One of the population groups affected is the indigenous populations, where sociodemographic and economic factors make them more vulnerable to these health problems.

Training (on knowledge and skills) is an important measure for tackling mortality rates, and especially training in multidisciplinary teams. This should certainly be put into practice in rural areas where traditional midwives play an important role, as they are the first contact of a large number of pregnant women in their communities. Currently, main barriers are mis-communication, and the lack of clarity of roles during patient care [21]. According to number 30 of the Joint Commission on the prevention of mortality and injury in childbirth, it is mentioned that some of the strategies that reduce the risk of adverse events are the following: review of the guidance and training process (70%), education and counseling (36%), review and reinforcing the protocols in a chain of communication (36%) [22]. Our study shows that PAR as a bottom-up approach can be a useful tool to improve local policies in obstetric emergencies. A current problem at the community level is lack of training and continuing education for traditional midwives; it is necessary to qualify them for giving care taking into account their knowledge and culture. In a study conducted in 1999 by the Medical Care Committee, it was estimated that medical errors are responsible for up to 98,000 deaths in the United States, and that one of the strategies to reduce these errors is individual and team training [23].

Among hypertensive disorders, pre-eclampsia is a main cause of maternal death, and its management in rural areas is complex due to the absence of a multidisciplinary team and to limited accessibility to the health care system. In rural environments, it is necessary to implement community interventions such as the one presented in this paper, which is focused on the midwives recognizing the warning signs of obstetric emergencies, making adequate decisions, and referring their patients to the (biomedical) health care system when needed. This study is consistent with two clinical trials, whose objective was to reduce, through community-based interventions, pregnancy adverse outcomes associated with delays in severity assessment, transportation and treatment [20, 24].

In our study, the midwives acknowledge that pre-eclampsia is an emergency that can compromise the mother’s and the child’s lives. Therefore, they look for warning signs and symptoms such as headache, lower limb edema and abdominal pain. These signs are in line with those indicated in the pre-eclampsia care guides from Gestational Hypertension and Preeclampsia: American College of Obstetricians and Gynecologists (ACOG) [25]. Although they have similar warning signs, not measuring blood pressure is a shortcoming. It causes a delay in diagnosis and thus, if resolved, could improve the care provided by midwives. Pre- and perinatal care means monitoring the patients for warning signs and symptoms and timely recognition of an obstetric emergency, which prevents potentially fatal complications [26]. The midwives interviewed as part of this study mentioned that they apply perinatal massages at the level of the uterine fundus until it descends below the navel. Subsequently, they apply a bandage to the patient so that the uterus remains contracted. In addition, they pay attention to the number of stained/bloody garments during delivery and postpartum and watch for signs such as pallor, low energy, somnolence, and palpitations, which may be associated with postpartum hemorrhage. Also, a number of delivery- and postpartum guides recommend watching the bleeding, the mentioned warning signs/symptoms and the massage at the level of the uterine fundus. In contrast to the work of midwives, oxytocin is used in the third stage of childbirth in biomedical health care system to reduce the risk of bleeding; midwives use herbs or oils [27, 28].

During delivery, one of the complications mentioned by the traditional midwives is “incorrect descent of the baby”, which can be due to a nuchal umbilical cord or to shoulder dystocia. One of the warning signs here is “*bebé chupado*” (“retracted baby”), known in the medical literature as the “turtle” sign. The midwives indicate that these complications are uncommon, and that vertical delivery care facilitates assistance in case they occur. On the other hand, the care provided in the biomedical health care system in this type of emergency requires training of the health personnel, such as the use of obstetric maneuvers as McRoberts [23, 29–31].

Limitations of our study include the fact that the perspective of the nationalities was not investigated from other cosmoviews. Furthermore, in the evaluation we focused on self-reported knowledge construction rather than on patient outcomes. Our sample did not allow us to study actual health improvement.

The use of PAR methodology was one of the strengths as it takes into account cultural, behavioural and contextual factors which hinder the application of research findings and guidelines [32]. Rather than doing research on a target group, in this case traditional midwives, it means to work together with them [33]. Developing a partnership in which different views on health and illness are recognized, is crucial for a successful collaboration [34]. It contributes to mutual trust and respect, which has shown to be an important condition to build continuity between traditional healers and the formal health system, as we observed in previous research in this population [35]. Also, it promoted program “buy-in” by the midwives, which was likely to contribute to the overall success of this project. Without attaining buy-in, willingness to participate in the program would be reduced, and even minor issues with the program could have resulted in midwives rejecting it, whereas, with buy-in, midwives are more likely to address and work through any unforeseen problems.

The researchers, representing the biomedical health system, and traditional midwives jointly decided to adapt the flowchart to the culture so that the method would be realistic and effective.

## Conclusion

Maternal mortality is still a severe problem, especially in indigenous and rural populations. In multi-ethnic countries where the health systems must be anchored with the different types of medicine, alternatives must be sought to reach the entire population, eliminating barriers such as miscommunication, hierarchization or intimidation across the different health care providers, inadequate follow-up of patients and geographical barriers.

Early recognition of the warning signs and symptoms in pregnant women is a priority. Training offers the necessary tools to improve care quality and reduce the number of complications during pregnancy, delivery and postpartum. Therefore, culturally adapted flowcharts were developed for the midwives to have a tool that allows them to provide more relevant care and to classify obstetric emergencies early, thus improving the clinical response.

## Data Availability

The datasets generated and/or analysed during the current study are not publicly available due to confidentiality of the participants but are available from the corresponding author on reasonable request.

## Abbreviations

PAR: Participant-Action Research
Kic: kichwa
M: Mixed race
Sh: shuar
ACOG: American College of Obstetricians and Gynecologists
MSP: Ministry of Public Health
